# Ectopic Overexpression of a Novel R2R3-MYB, *NtMYB2* from Chinese Narcissus Represses Anthocyanin Biosynthesis in Tobacco

**DOI:** 10.3390/molecules23040781

**Published:** 2018-03-28

**Authors:** Muhammad Anwar, Guiqing Wang, Jiacheng Wu, Saquib Waheed, Andrew C. Allan, Lihui Zeng

**Affiliations:** 1 College of Horticulture, Fujian Agriculture and Forestry University, Fuzhou 35002, China; anwar_uaar@yahoo.com (M.A.); guiqingw@hotmail.com (G.W.); liablo434@outlook.com (J.W.); gulanwar85@gmail.com (S.W.); 2 The New Zealand Institute for Plant & Food Research, Mt Albert Research Centre, Private Bag 92169, 1142 Auckland, New Zealand; andrew.allan@plantandfood.co.nz; 3 School of Biological Sciences, University of Auckland, Private Bag 92019, 1142 Auckland, New Zealand

**Keywords:** Chinese narcissus, R2R3 MYB, anthocyanin repressor, anthocyanin pathway, transcriptional regulation

## Abstract

R2R3 MYB transcription factors play key functions in the regulation of secondary metabolites. In the present study, a R2R3 MYB transcriptional factor *NtMYB2* was identified from Chinese narcissus (*Narcissus tazetta* L. var. *Chinensis* Roem) and functionally characterized. *NtMYB2* belongs to subgroup 4 of the R2R3 MYB transcription factor family that are related to repressor MYBs involved in the regulation of anthocyanin and flavonoids. Transient expression confirmed that *NtMYB2* strongly reduced the red pigmentation induced by MYB- anthocyanin activators in agro-infiltrated tobacco leaves. Ectopic expression of *NtMYB2* in tobacco significantly reduced the pigmentation and altered the floral phenotypes in transgenic tobacco flowers. Gene expression analysis suggested that *NtMYB2* repressed the transcript levels of structural genes involved in anthocyanin biosynthesis pathway, especially the *UFGT* gene. *NtMYB2* gene is expressed in all examined narcissus tissues; the levels of transcription in petals and corona is higher than other tissues and the transcription level at the bud stage was highest. These results show that *NtMYB2* is involved in the regulation of anthocyanin biosynthesis pathway and may act as a repressor by down regulating the transcripts of key enzyme genes in Chinese narcissus.

## 1. Introduction

Chinese narcissus (*Narcissus tazetta* L. var. *chinensis* Roem) is a perennial bulbous plant which belongs to the Amaryllidaceae family. It has very popular attractive ornamental flowers with significant commercial and cultural value. It has been grown in China and East Asia from many years as a traditional and commercial flower. Narcissus has been reported as a triploid species, mostly propagated by a vegetative method, as genetically advanced improvement using traditional cross breeding is complicated [1]. Therefore, only a few cultivars have been used for commercial production in China and many agronomic characters have to be improved such as flower colors [2]. The flower color and pigmentation patterns are the very important consideration for consumer choice. In Chinese narcissus, the flower colors are white and yellow. The compositions of the flavonoid compounds in narcissus cultivars were analyzed, and found to be flavonols, with no anthocyanins detected [3]. Therefore, the investigation on the molecular mechanisms of why narcissus has no anthocyanins biosynthesis could provide benefit for developing new cultivars with diverse color patterns.

Flavonoids are a group of diverse secondary metabolites which are broadly distributed in plants consisting of the flavones, isoflavones, flavonols, anthocyanins and proanthocyanidins (PAs). Anthocyanins are reported as common natural plant pigments which contribute different colors such as blue, purple and red to the fruits, vegetables and flowers [4]. In plants, key genes encoding enzymes involved in anthocyanin pathway have been identified. Chalcone is considered as the general precursor of flavonoid compounds that comprise of coumaroyl-CoA and malonyl-CoA. Leucoanthocyanidins is derived from coumaroyl-CoA and malonyl-CoA by chalcone synthase (*CHS*), chalcone isomerase (*CHI*), flavanone 3-hydroxylase (*F3H*) and dihydroflavonol reductase (*DFR*) [5]. Finally, anthocyanins and PAs products are obtained by the activity of anthocyanin synthase (*ANS*). Anthocyanidin reductase leads to the end product of catechin while epicatechin is derived from leucoanthocyanidin reductase, both are related to proanthocyanin biosynthesis pathway [6,7,8].

Flavonoid biosynthesis is regulated by several transcriptional factors, including R2R3 MYB, basic helix-loop-helix (bHLH) and WD40 repeat (WDR) proteins [9]. These TFs act together as a MYB-bHLH-WDR (MBW) complex to activate the structural genes which are involved in flavonoid biosynthesis pathway. These TFs have been functionally characterized in many plants including maize, Arabidopsis and soybean, strawberry, petunia, apple, grapevine and populous [10,11,12,13,14,15,16].

In plants, many R2R3 MYB transcriptional factors have been identified as activators of flavonoid biosynthesis, for example, *PAP1* is involved with the modulation of anthocyanin and *TT2* regulates the proanthocyanidin biosynthesis in *Arabidopsis* [17,18]. In grapevine, *VvMYBA1* and *VvMYBA2* are involved in the activation of anthocyanin biosynthesis by modulating *UFGT* gene expression [19]. *VvMYBA1* is also involved in the regulation of PA production by stimulating *LAR* and *ANR* expression [20]. In apple, *MdMYB1* is associated in the biosynthesis of anthocyanin in apple fruit skin [21].

R2R3 MYB proteins are more conserved in the *N*-terminal DNA binding domains while protein sequence at the *C*-terminus are divergent and believed to be responsible for several kinds of regulatory functions [22]. Based on specific conserved regions in the *C*-terminus, R2R3 (MYBs TFs) have been classified into 22 subgroups [23]. Subgroup 4 R2R3-MYB transcriptional factors function as negative regulators in phenylpropanoid and flavonoid biosynthesis. *VvMYB4-like* from grapevine, *PhPH4*, *PhMYB27* from petunia, *MdMYB16* from apple, *GmMYB100* from soybean, *PtrMYB57*, *PtrMYB182* from poplar, *AtMYB60* from *Arabidopsis*, *SsMYB3* from coleus reduces the accumulation of anthocyanin by suppressing the key anthocyanin biosynthesis pathway genes [16,24,25,26,27,28,29,30,31]. *CmMYB1* from chrysanthemum, *AtMYB7* from *Arabidopsis*, *FaMYB1* from strawberry reduced the flavonol biosynthesis [10,32,33].

In addition, ectopic expression of *AmMYB308* and *AmMYB330* from *Antirrhinum majus* in tobacco plants negatively affects phenolic acids and lignin biosynthesis [34]. Overexpression of *ZmMYB31* and *ZmMYB42* in *Arabidopsis* reduced the content of lignin in *Arabidopsis* [35,36]. *AtMYB4* and *AtMYB32* are involved in the regulation of flavonol, sinapate esters and lignin biosynthesis [37,38]. In addition to R2R3 MYB repressors in Subgroup 4, single-repeat R3-MYB proteins also have been identified which are involved in the negative regulation of anthocyanin accumulation, including *ETC1*, *AtMYBL2* and *MYBx* from *Arabidopsis* and *Petunia* [39,40]. *AtMYBL2* has a *C*-terminal TLLLFR motif, which also contributes to suppressive activities [41].

A lack of anthocyanins may be due to inhibition of the anthocyanin biosynthesis pathway. Transcriptional factors involved in the downregulation of anthocyanin or flavonoid biosynthesis have not been identified in narcissus. The present investigation on molecular mechanisms controlling flower color in narcissus would provide beneficial breeding programs that look to develop new cultivars with diverse color patterns. In present study, a putative *R2R3-MYB* gene, *NtMYB2* was cloned from *Narcissus*. It was functionally characterized in detail by bioinformatics analysis, transient co-transformation, qPCR analysis and ectopic expression in tobacco. The results suggest *NtMYB2* may be a repressor of anthocyanin biosynthesis. The putative role of *NtMYB2* will provides new approaches to understanding the regulator mechanisms of flavonoid biosynthesis in *Narcissus* spp.

## 2. Results

### 2.1. Cloning of NtMYB2

A partial sequence (769 bp) of a R2R3-MYB gene was obtained from transcriptome data and named *NtMYB2*. The 3′ end of *NtMYB2* ORF was isolated by 3′ RACE-PCR with two gene specific primers GP1, GP2 (Table 1) and universal primer (UPM) and a 912 bp fragment was obtained. The full length of *NtMYB2* ORF -was 756 bp encoding 251 amino acids residues with theoretical pI 7.95 and molecular weight of 28.3 kDa was obtained.

### 2.2. Subcellular Localization

Insilico investigation for subcellular localization (Available online: http://nls-mapper.iab.keio.ac.jp/cgi-bin/NLS_Mapper_form.cgi) showed the existence of nuclear localization signal “RPDLKRGNFTEEEDDLIIKLHGM” starting from amino acid at 62th location, suggesting nuclear localization of NtMYB2.

### 2.3. Sequence Analysis and Phylogenetic Tree

**Figure 1:**
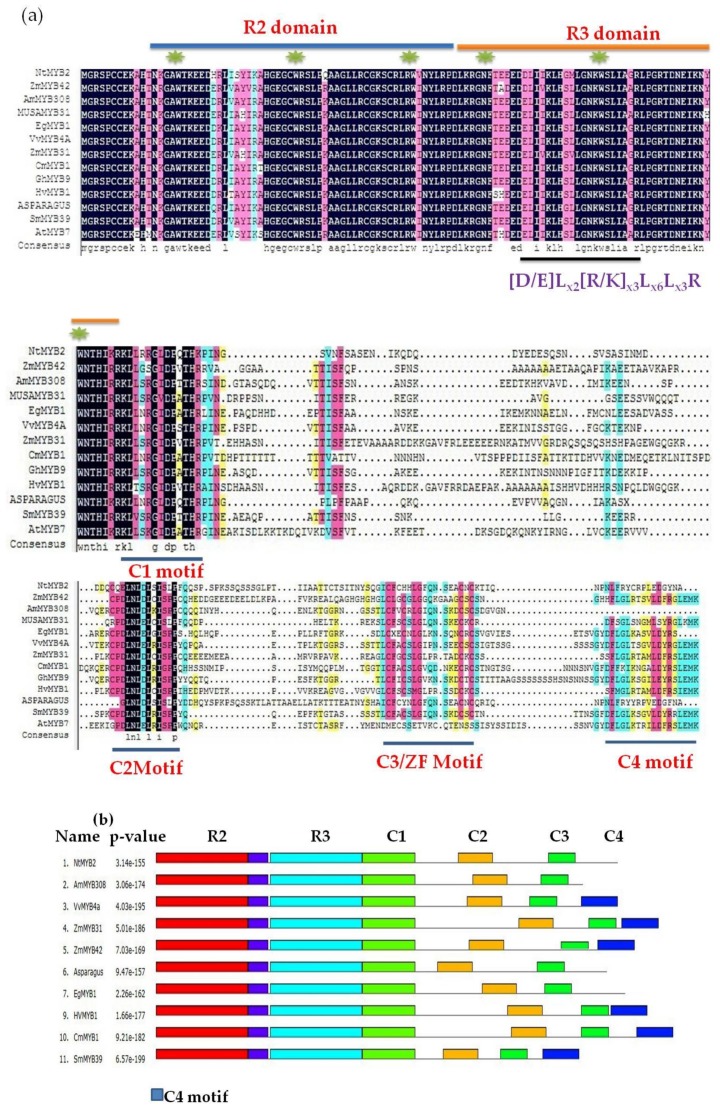

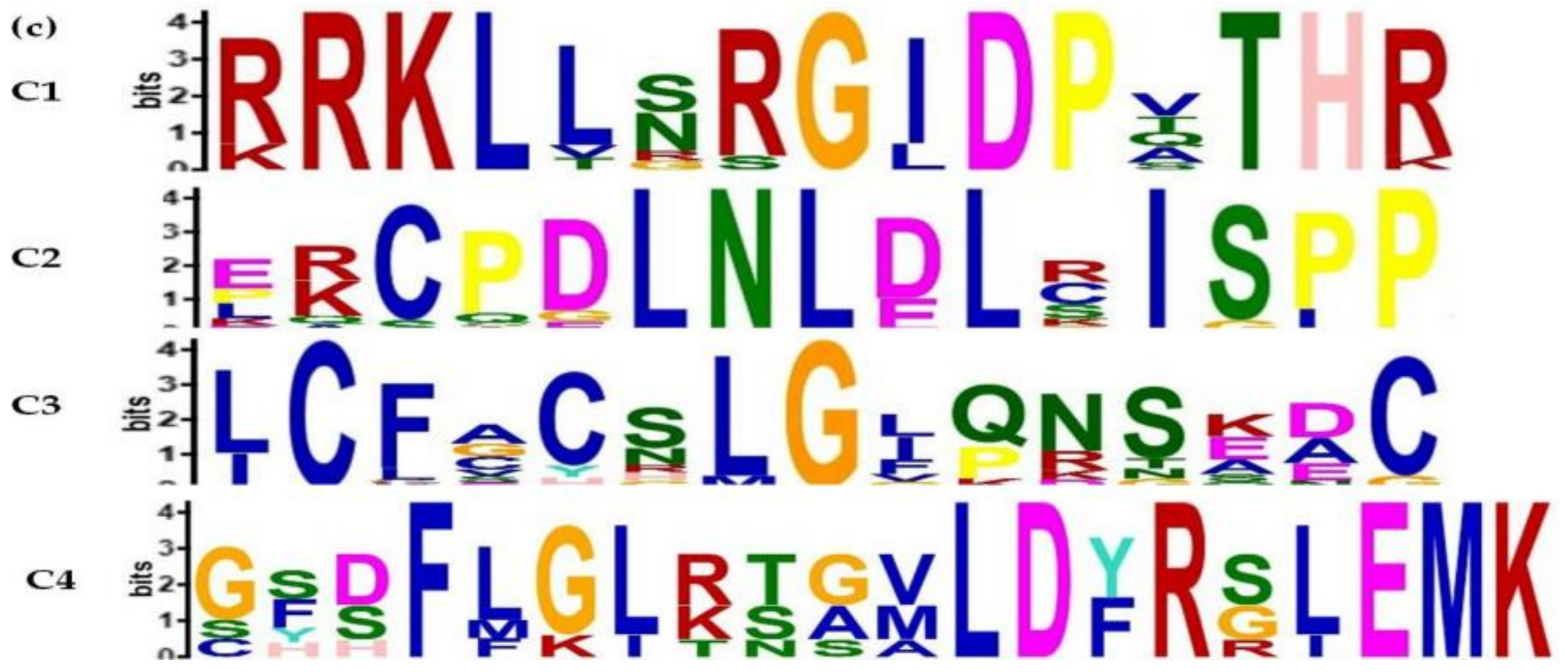
(**a**) Multi -alignment of the amino acid sequence of *NtMYB2* with other repressor R2R3 MYB of subgroup 4. The R2 and R3 binding domain are indicated by blue and gray colored lines respectively on top. A black underline within the R3 domain shows the motif (consensus sequence [DE]Lx_2_[RK]x_3_Lx6Lx_3_R) interacting with the bHLH protein. The conserved amino acids, tryptophan (W) and phenylalanine (F) are shown by the green star. The C1 and C2 functional motifs are underlined in the blue line. The C3/ZF like and C4 motif are also underlined with blue color. Sequences utilized for alignment are: *Narcissus tazetta* L. var. *chinensis* NtMYB2 (KY860527.1). *Antirrhinum majus* AmMYB308 (P81393), *Vitis vinifera* VvMYB4a (XP_002278222), *Gossypium hirsutum* GhMYB9 (AAK19619), *Hordeum vulgare* HvMYB1 (P20026), *Chrysanthemum morifolium* CmMYB1 (AEO27497), *Arabidopsis thaliana* AtMYB7 (NP_179263), *Salvia miltiorrhiza* SmMYB39 (KC213793), *Eucalyptus gunnii* EgMYB1 (CAE09058.1), *Asparagus officinalis* (XP_020246745) and *Zea mays* ZmMYB42 (NP_001106009.2), ZmMYB31 (NP_001105949.2), (**b**) MEME motif logo with *p*–value, (**c**) MEME motif logos with bit scores.

**Figure 2:**
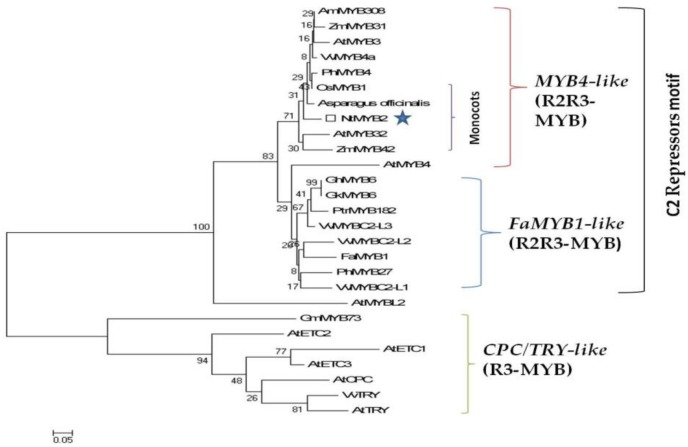
Phylogenetic tree analysis of MYB repressors based on amino acid sequences consisting R2R3 domain or R3 domains. Bar 0.05 substitutions per site. The accession numbers of MYB proteins used in tree building are: *Zea mays* ZmMYB42 (NP_001106009.2), *Zea mays* ZmMYB31 (NP_195225.1), *Vitis vinifera* VvMYB4a (ABL61515.1), *Arabidopsis thaliana* AtMYB32 (NP_195225.1), AtMYB3 (NP_564176.2), AtMYB4 (AAC83582.1), AtMYBL2 (AEE35154), *Petunia hybrida* PhMYB4 (ADX33331.1), PhMYB27 (AHX24372.1), *Fragaria ananassa* FaMYB1 (AAK84064.1), *Vitis vinifera* VvMYBC2-L1 (ABW34393.1), VvMYBC2-L2 (ACX50288.2), VvMYBC2-L3 (AIP98385.1), *Antirrhinum majus* AmMYB308 (P81393), *Oryza sativa* OsMYB1 (AK104457), *Gossypium hirsutum* GhMYB6 (AAN28286), *Glycine max* GmMYB73 (ABH02868), VvTRY (ABW34395), *Arabidopsis thaliana* AtTRY (Q8GV05), CPC (BAA21917), ETC1(NP_171645), (ETC2 AEC08385), ETC3 (AEE81974), *Gossypioides kirkii* GkMYB6 (AAN28289), *Populus tremuloides* PtrMYB182 (Potri.004G088100).

Sequence analysis showed that NtMYB2 contained conserved R2R3 domain at the *N*-terminal end. In addition, NtMYB2 protein has the conserved motif of [D/E]Lx_2_[R/K]x_3_Lx_6_Lx_3_R in the R3 domain accountable for interaction with R like bHLH protein. NtMYB2 protein also contained the conserved motif of L17x3L21x2R24 in the R3 domain (Figure 1a). Comparisons of NtMYB2 with other R2R3 MYB transcriptional factors indicated that NtMYB2 show high homology with AoMYB (73.05%, similarity) in asparagus, MUSAMYB31 (56.75% similarity) in banana, VvMYB4a (55.64%, similarity) in grapevine AmMYB308 (55.38%, similarity) in antirrhinum and PhMYB4 (54.75%, similarity) in Petunia. These genes belong to the subgroup 4 of R2R3 MYB transcriptional factors which consist of four conserved motifs at *C*-terminal region. These conserved motif are called C1 motif (KLIsrGIDPxT/SHRxI/L), C2 motif (pdLNLD/ELxiG/S), C3 or ZnF (zink-finger) like motif (CX1-2CX7-12CX2C) and C4 motif (FLGLx4-7V/LLD/GF/YR/Sx1LEMK) [12,42]. C2 repressor motif associated with EAR repressor domain [37,43]. The analysis of protein sequence revealed that NtMYB2 shows the LxLxL type EAR motif with others subgroup 4 R2R3 MYB transcriptional factors such as VvMYB4a and VvMYB4b, AtMYB7 and AtMYB32 which are involved in the suppression of flavonoids and lignin biosynthesis pathway [43] (Figure 1a). However, NtMYB2 lacks C4 motif unlike other members of subgroup 4 R2R3 MYB proteins (Figure 1b,c). The phylogenetic analysis indicated that R2R3 MYB TFs that consist of C2 repressor motif were divided into two clades such as AtMYB4-like and FaMYB1-like. Single- repeat R3-MYB protein containing repressor CPC/TRY like clade was separated. *NtMYB2* was grouped within MYB4- like R2R3 MYB TFs clade (Figure 2), which is concerned for transcriptional repression including *PhMYB4* from Petunia, *VvMYB4a*, from the grapevine, AmMYB308 from *Antirrhinum*, AtMYB32. AtMYB4, AtMYB3 from *Arabidopsis*, ZmMYB31 and ZmMYB42 from Maize, AoMYB from asparagus, OsMYB1 from rice. NtMYB2 also has the close relationship with other MYB TFs from monocot plants such as rice, maize and asparagus (Figure 2). On the basis of sequence alignments and phylogenetic analysis, NtMYB2 may act as a transcriptional repressor of flavonoids biosynthesis pathway.

### 2.4. The Expression Pattern of NtMYB2 in Chinese Narcissus

**Figure 3:**
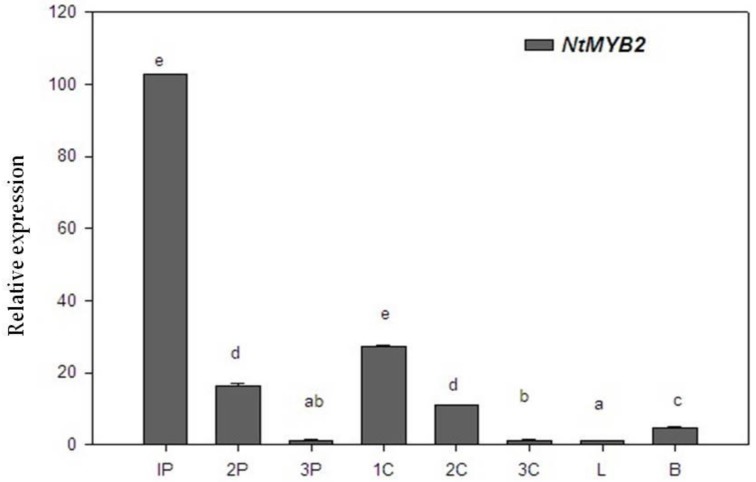
The expression pattern of NtMYB2 in Chinese Narcissus.

The expression profile of *NtMYB2* was examined in different tissues of Chinese narcissus. Quantitative RT-PCR results indicated that *NtMYB2* was expressed in all types of tissues examined. The expressions in petals and corona was significantly higher than in basal plates and leaves. Flower petals at the bud stage showed a relatively higher expression as compared to petals at half opened and full opened stage. The similar trend of expression levels was observed in corona (Figure 3).

1P, 2P and 3P indicate the different developmental stages of petals at bud stage, half opened stage and full stage respectively. 1C, 2C and 3C denote for corona different developmental stages as bud stage, half opened stage and full opened stage. L and B represent for leaves and basal plates respectively. The bars indicate the standard error of three biological replicates. Letter represents a significant difference at the level of *p* < 0.05 using LSD statistical analysis.

### 2.5. Transient Expression of NtMYB2 in Tobacco

**Figure 4:**
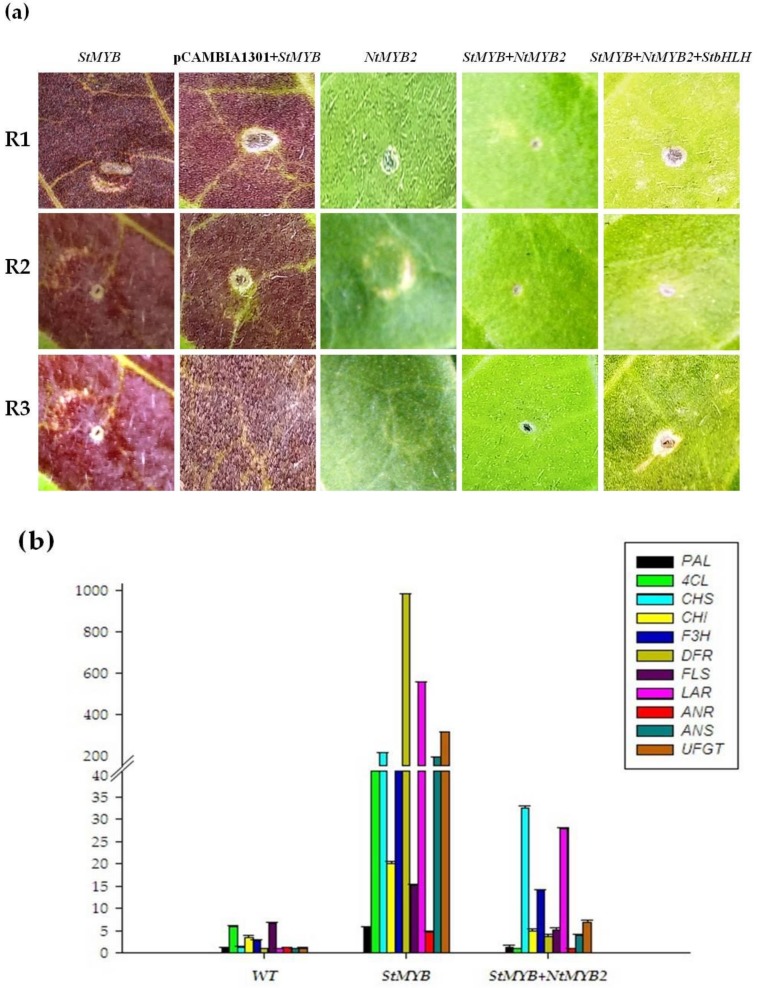
Transient transformation of *Nicotiana tabacum* leaves. (**a**) Color changes in agroinfiltrated leaves of *Nicotiana tabacum*. R1, R2 and R3 indicated three replicates. (**b**) Expression analysis of flavonoid biosynthesis pathway genes in agroinfiltrated leaves of *Nicotiana tabacum* by qPCR.

In order to functionally characterize *NtMYB2*, transient assays were carried out in *Nicotiana tabacum* through *Agrobacterium* infiltration method as previously report [44]. Agroinfiltration with different treatments were performed. Visible red pigmentation was observed with syringe-infiltrated *StMYB* (potato *StAN1*, positive control) [45]. Interestingly, red pigmentation disappeared in tobacco leaves co-infiltrated with *StMYB* and *NtMYB2*. No red pigmentation or anthocyanin patch was observed with *NtMYB2* infiltrated by itself. There was also no observed induction of anthocyanin pigmentation in leaves co-infiltrated with 3 genes of *NtMYB2*, *StMYB* and *StbHLH* (Figure 4a).

In order to verify the effect of *NtMYB2* on the expression levels of the structural genes which are involved in the biosynthesis of flavonoid pathway in syringe infiltrated tobacco leaves, the expression of *PAL*, *4CL*, *CHS*, *CHI*, *F3H*, *FLS*, *DFR*, *ANR*, *LAR, ANS* and *UFGT* were assessed by qRT-PCR. The expression levels of all these structural key genes except for *PAL* and *ANR* were significantly induced in the injected leaves with single *StMYB* and were significantly reduced in co-infiltrated leaves with *NtMYB2* and *StMYB* (Figure 4b).

### 2.6. Stable Expression of NtMYB2 in Tobacco

**Figure 5:**
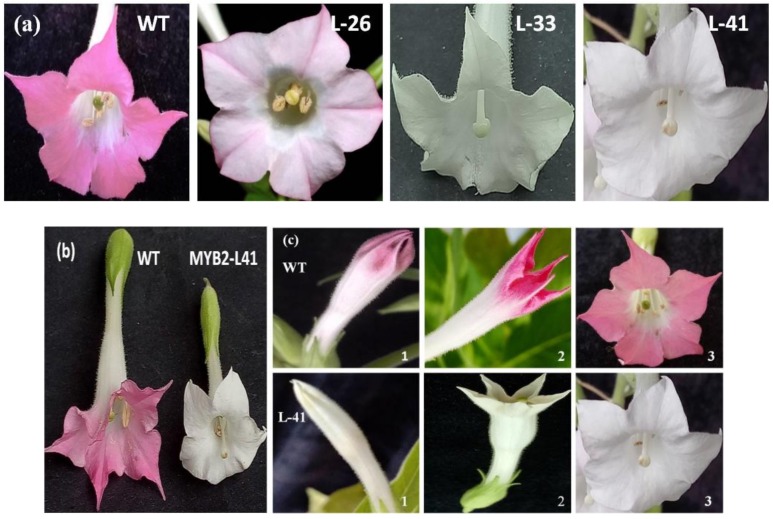
Floral phenotypes of transgenic tobacco plants. Floral phenotypes of transgenic tobacco plants over expressing *NtMYB2* gene. WT, wild type tabacco; L-26, L-33 and L-41, three transgenic tobacco lines. (**a**) Comparison of flower color in transgenic tobacco flowers and WT; (**b**) Comparison of flower size and pistil length in WT and transgenic tobacco; (**c**) Comparison of flower color of in transgenic tobacco flowers and WT at different flower stages. 1-bud stage, 2-half opened stage, 3-full open stage.

**Figure 6:**
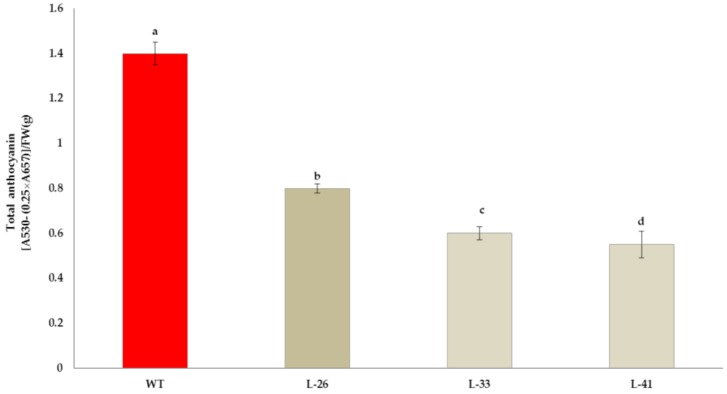
Quantification of total anthocyanin. Quantification of total anthocyanin in flowers of *NtMYB2* tobacco lines (L-26, L-33) and wild type (WT). The bars indicate the standard error of three biological replicates. Letter represents a significant difference at the level of *p* < 0.05 using LSD statistical analysis.

*NtMYB2* under the expression of the 35S promoter was transformed into tobacco. The existence of introduced *NtMYB2* was confirmed by PCR (Figure S1). Transgenic tobacco plants didn’t show any growth changes. The transgenic plant flowers showed visible phenotypic changes in petal pigmentation patterns; from light pink to almost white. In particular, the transgenic tobacco Line-41 (L-41) had a significant change of flower color. The L-26 showed very pale pink flowers with pale red veins and the edge of petal (Figure 5a). In addition to the flowers color, the length of pistil of transgenic flowers was elongated, compared to that of the WT flower. Furthermore, the flowers size was smaller as compared to the control. The stigma color of transgenic lines turned yellow and a green to slightly green color was noticed in the sepals of transgenic flowers (Figure 5b). The transgenic tobacco flowers at early developmental stage showed no pigmentation, while in wild type tobacco flowers visible pigmentation were observed (Figure 5c). These results showed that ectopic overexpression of *NtMYB2* in tobacco leads to decrease the accumulation of anthocyanin to alter the flower color in transgenic flowers. Quantitative analysis confirmed the reduction of anthocyanin levels in the flowers of transgenic plants (Figure 6).

### 2.7. Overexpression of NtMYB2 in Tobacco Influences the Expression Levels of Flavonoid Biosynthetic Pathway Genes

**Figure 7:**
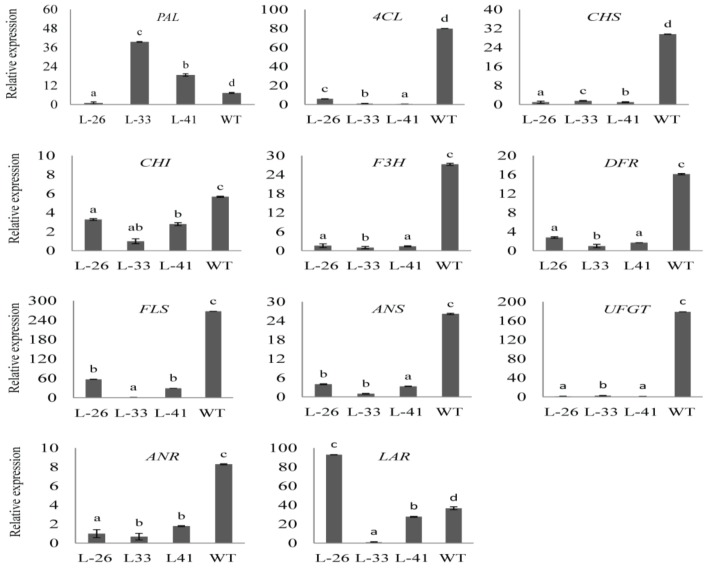
Expression analysis of flavonoid biosynthesis pathway genes. Expression analysis of flavonoid biosynthesis pathway genes in flowers of *NtMYB2* transgenic tobacco. L-26, L-33, L-41 means three transgenic line. The bars indicate the standard error of three biological replicates. Letters (a, b, c and d) represent the significant difference from wild type at the level of *p* < 0.05 using LSD test.

The effect of overexpression of *NtMYB2* on the transcript levels of flavonoid biosynthetic pathway genes in transgenic tobacco flowers was assessed by qRT-PCR assay. The analysis of qPCR results indicated that tobacco anthocyanin biosynthetic genes including *CHS*, *CHI*, *F3H*, *DFR*, *ANS*, and *UFGT* were significantly down-regulated in *NtMYB2-26, NtMYB2-33* and *NtMYB2-41* overexpression lines compared to the control tobacco flowers (Figure 7). The transcript levels of *FLS* and *ANR* were also significantly down-regulated in three overexpression lines. The *LAR* expression was down-regulated in the *NtMYB2-33* and *NtMYB-41* overexpression lines but was up regulated in the *NtMYB2-26* overexpression line as compared to the control. The transcript level of *PAL* which was involved in general phenylpropanoid pathway was up-regulated in transgenic tobacco Lines 33 and 41. These results showed that ectopic expression of *NtMYB2* transcriptional factors significantly down-regulates the expression levels of main flavonoid biosynthetic genes in tobacco.

## 3. Discussion

R2R3-MYB transcriptional factors are a large gene-family in plants and have been reported to play key roles in the regulation of secondary metabolites. In the current study, we identified a novel R2 R3 MYB transcriptional factor from narcissus, termed as *NtMYB2*, which may repress anthocyanin biosynthesis in transgenic tobacco as well as in narcissus.

### 3.1. Bioinformatics Analysis Showed NtMYB2 Was a Transcriptional Factor

Flavonoid biosynthesis is regulated by MBW complexes, among which MYB proteins that consist of activators and repressors. The amino acid sequence of NtMYB2 showed high homology with other R2R3 -MYB proteins from plants, such as AtMYB7, AmMYB308, ZmMYB42, VvMYB4 and CmMYB1 [25,32,33,34,46]. These are implicated in the negative regulation of flavonoid biosynthesis. Further protein motif analysis indicated that NtMYB2 contained typically three conserved motifs as C1, C2 and C3 motif at C-terminal. Subgroup 4 R2R3-MYB was initially defined by the existence of C1, C2, and C3 motifs [12]. Members of subgroup 4 R2R3-MYB proteins have C2 repressor motif which is responsible for repression activity in flavonoid biosynthesis pathway genes [47,48,49,50]. The results of NtMYB2 protein analysis showed the sequence to be similar to PtrMYB57 and VvMYB4- like, both have C2 motif and are involved in the suppression of anthocyanin accumulation [25,28]. The presence of a conserved C2 motif sequence “pdLNLD/ELxiG/S” suggests that NtMYB2 may be act as a repressor of flavonoid biosynthesis. However, NtMYB2 lacks the C4 (dFLGL and LDF/YRxLEMK) motif and showed divergence in the C-terminus from other members of subgroup 4. The C4 motif is also absent in snapdragon AmMYB308, which is known as a flavonoid repressor [34]. In addition, we have found the existence of bHLH interaction residues that are present in R3 repeat of NtMYB2. Theses residues indicate the conserved region ([DE]Lx_2_[RK]x_3_Lx6Lx_3_R) interacting with the bHLH proteins as described in *Arabidopsis* [51]. NtMYB2 protein also contained the conserved motif of L17x3L21x2R24 in the R3 domain and supposed to stabilize the interaction of bHLH-MYB proteins [16]. Phylogenetic analysis revealed that NtMYB2 was grouped in MYB4-like clade with VvMYB4a, PhMYB4, and CmMYB1. In Subgroup 4, AtMYB4, AtMYB3 from Arabidopsis, VvMYB4a and VvMYB4b from grapevine and PtrMYB182, PtrMYB57 from poplar are associated with down-regulation of the anthocyanin and PA biosynthesis [12,37]. AtMYB7 in subgroup 4 acts as a repressor of flavonol biosynthesis [33]. The results of bioinformatics analysis showed that NtMYB2’s function may be similar with other MYBs in subgroup 4, acting as a transcriptional repressor. R2R3 MYB repressors may have two kinds, one of which functioned on MBW complexes such as FaMYB1 and the other directly binds on target genes such as *AtMYB4* [27]. NtMYB2 is grouped with MYB4-like clade indicates this gene may directly bind on target genes consistent with the results of MdMYB16 from apple which directly binds on *ANS* and *UFGT* that inhibit the anthocyanin biosynthesis [27].

Effective accumulation of anthocyanin biosynthesis by the *MdMYB10* is dependent on the co-expression of a specific bHLH protein. In *Petunia*, PhAn1, a *bHLH* transcriptional factor directly induces the expression of the biosynthetic gene *DFR* and is controlled by MYB factor *PhAn2* [52]. This difference may be due to the characteristic of the exogenous MYBs and the interactions with endogenous tobacco bHLH factors. The previous study showed that the *PamMYBA.1* and *PamMYBA.2* regulate the anthocyanin biosynthesis in citrus and tobacco without requiring any further cofactors. While *PamMYBA.3* required the additional cofactors (*bHLH*) for the induction of anthocyanin accumulation [53]. These results indicate that some MYB transcriptional factors need co-expression of the bHLH simultaneously to perform functional activates, while other MYB transcriptional factors alone are sufficient to induce or reduce the accumulation of anthocyanin.

### 3.2. NtMYB2 Reduced the Pigmentation in Tobacco Leaves with Transient Assay

*StMYB* is from potato, which is involved in positive regulation of *DFR* gene [45]. When *StMYB* was agro-infiltrated in tobacco leaves alone, it induced obvious red pigmentation on leaves (Figure 4)*.* A significant loss of red pigmentation was observed in co-injected leaves with *StMYB* and *NtMYB2* indicating *NtMYB2* represses anthocyanin biosynthesis. qPCR analysis showed that in injected leaves co-infiltrated with *NtMYB2* and *StMYB,* the transcript levels of key anthocyanin biosynthesis structural genes (*CHS*, *CHI, F3H*, *DFR, ANS* and *UFGT*) were significantly down-regulated. Previous study has shown that poplar *PtrMYB57* reduced the transcript level of flavonoid biosynthetic genes including *4CL*, *CHS*, *DFR*, *ANS*, *ANR* and *LAR* leading to the repressing of anthocyanins and *PAs* in tobacco leaves [28]. The evidence continued repression in the presence of *StbHLH* co-infiltration indicated that *NtMYB2* may not be competing with for *StbHLH* with *StMYB* and may play a role by directly binding with the promoters of structural genes.

### 3.3. NtMYB2 Reduces the Flower Color of Transgenic Tobacco

Ectopic expression of *NtMYB2* in tobacco repressed the pigmentation in petals of transgenic tobacco flowers. In addition, a change in stigma color was observed in some overexpression lines. Phenotypic changes were only observed in flowers, not in vegetative parts. This present study agrees with the results of *VvMYB4-like* transcriptional regulators [25]. Overexpression of *VvMYB4-Like* in tobacco lead to the loss of pigmentation in flowers due to the reduction in anthocyanin accumulation [25]. Lower anthocyanin levels in *NtMYB2* overexpression lines correlated with the observed loss of pigmentation. *PtrMYB57* (Subgroup 4) that reduced the contents of anthocyanin in transgenic poplar plants than wild type [28]. Overexpression of *Coleus SsMYB3* significantly reduced the anthocyanin contents in all transgenic tobacco plants and also strongly decreased the color pigmentation of transgenic flowers than those of wild type [31]. In grape, *VvMYBPA1* showed significantly lower amounts of anthocyanin and reduction of petal pigmentation in transgenic tobacco flowers as compared to flowers of wild type plants [54]. These support the hypothesis that *NtMYB2* is an anthocyanin repressor.

In addition to a loss of flower pigmentation, differences in other morphological characters, such as smaller shorter flower were seen in *NtMYB2* lines compared to wild type. Our findings are similar with previous studies, when *AmMYB308* from *Antirrhinum* is over expressed in transgenic tobacco, flower size and the level of anthocyanin are reduced as compared to wild type [34]. Furthermore, the length of pistils of *NtMYB2* overexpression lines were elongated compared to wild type, with stigmas being above the anther. Similar results have been reported for transgenic tobacco lines expressing the apple *MdMYB3* gene [55]. How these MYB repressors induce other morphological character changes needs further investigation.

### 3.4. NtMYB2 Is Involved in Regulation of Anthocyanin/Flavonoid Biosynhesis Patway Genes in Tobacco

**Figure 8:**
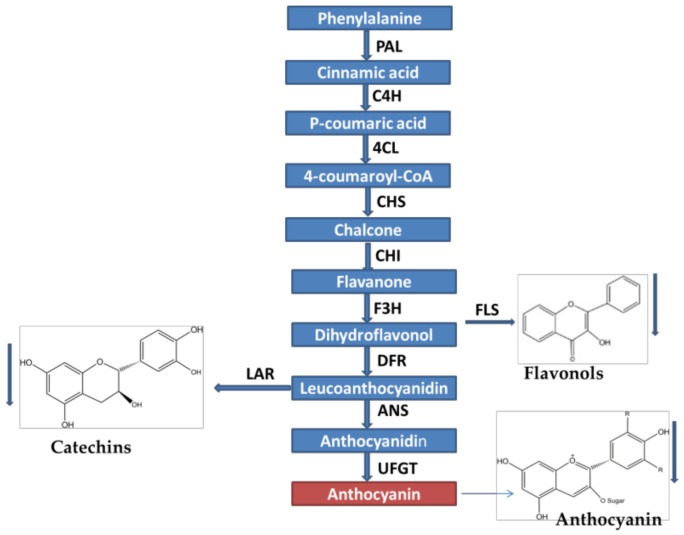
General flavonoid biosynthesis pathway. Arrow indicating the decreasing flavonol, catechins and anthocyanin biosynthesis pathway in transgenic flower of tobacco lines.

The change of anthocyanin biosynthesis in transgenic tobacco flowers implied that the expressions of flavonoid biosynthetic genes were affected by *NtMYB2* overexpression (Figure 8). In transgenic tobacco flowers, all of anthocyanin pathway genes were significantly down-regulated, including *CHS*, *CHI*, *F3H*, *DFR*, *ANS* and *UFGT*. *VvMYB4-like* gene negatively regulates *ANR* and *FLS* gens in transgenic tobacco flowers [25]. Our results showed that *NtMYB2* down-regulates both early and late key anthocyanin biosynthesis pathway genes. In addition, *NtMYB2* down-regulated the proanthocyanidin (PA) biosynthetic pathway gene *NtANR* in tobacco flowers, while *NtLAR* showed variable expression levels in transgenic tobacco flowers. These results suggest *NtMYB2* may negatively regulate the catechin biosynthesis pathway. Moreover, the expression level of *NtFLS*, involved in flavonol biosynthesis, also decreased in *NtMYB2*-overexpression tobacco flowers.

The transcripts level of *UFGT* was significantly reduced in all *NtMYB2* overexpression tobacco lines as compared to the control plants. In grapevine *VvMYB4-like* and *VvMYB4A* reduced the anthocyanin by down regulating the *DFR*, *ANS* and *UFGT* genes respectively [25,56]. Ectopic expression of *SsMYB3* in tobacco significantly down-regulated the transcripts level of *NtUFGT* that leads to reduce the accumulation of anthocyanin [31]. Over expression of *FaMYB1* in tobacco reduce the contents of anthocyanin by reducing the expression level of *UFGT* [10].

### 3.5. Expression Profiles of NtMYB2 in Narcissus

The results of qPCR analysis showed that *NtMYB2* has expression in flowers, leaves and basal plates of narcissus. The transcript level of *NtMYB2* was much higher in petals and corona as compared to vegetative tissues, indicating that *NtMYB2* may play a repressive role in downregulating anthocyanin levels in flowers. This may be a key reason why no anthocyanin accumulates in flowers of narcissus. In addition, the expression of *NtMYB2* is affected by different developmental stages of flowers. In ornamental peach flowers, *PpMYB17* and *PpMYB20* repressors were significantly expressed at bud stages, but remarkably decreased at full open stages and showed decreasing expression pattern during flower development [57].

## 4. Materials and Methods

### 4.1. Plant Materials

The flowers, basal plate and leaf of Chinese narcissus were used for the RNA extraction and further experiments. The seeds of *Nicotiana tabacum* plants were grown in pots contain compost mix (1:1 compost: perlite and 3:1 compost: perlite for seed and plants, respectively) and maintained in the glasshouse. The young leaves of tobacco were used for transient expression. The flowers of transgenic tobacco were used for the qPCR analysis and also used for extraction of total anthocyanin and total phenolics contents.

### 4.2. RNA Extraction

Total RNA was extracted using the Universal Plant total RNA extraction Kit (Bioteke Corporation, Beijing, China), following the manufacturer’s instructions. RNase free DNase I treatment (Invitrogen, Waltham, MA, USA) was performed to eliminate contaminant and residual genomic DNA. The integrity of extracted RNA was checked on agarose gels stained with ethidium bromide. The RNA concentration was calculated in an ND-1000 UV spectrophotometer (Nanodrop Technologies, Wilmington, DE, USA). The cDNA first strand was synthesized from 1 µg total RNA using SMARTer^®^RACE 5′/3′ kit (Clontech Laboratories, Inc., Palo Alto, CA, USA).

### 4.3. 3′RACE of NtMYB2

The full-length cDNA of *NtMYB2* was obtained by extending the 3′end using SMARTer^®^RACE 5′/3′ kit (Clontech Laboratories, Inc.). The 3′end sequence was obtained by two rounds of PCR amplifications with gene specific primers and a universal primer (provided in the kit). Nested PCR involved the nested universal primer and the second gene specific primer. The first gene specific-primer was *NtMYB2-GSPF1* 5′-ATGGGTAAACTATCTTCGGC-3′ and the second gene-specific primer was *NtMYB2-GSPF2* 5′-GGAAGGACAGACAACGAGAT-3′. The total 50 µL PCR mixture (Ex Taq, Takara, Dalian, China) contained 5 µL buffer, 4 µL dNTPs, 1 µL DNA polymerase, 2 µL first strand cDNA, 0.5 µM each primer and 36 µL double distilled water. The PCR reaction was carried out as follows: pre-heating at 95 °C for 5 min, 34 cycles at 95 °C for 30 s, the annealing temperature was 57 °C for 1 min and 72 °C for 1 min, then an extension at 72 °C for 7 min. The PCR amplified fragment was purified and then cloned into pMD 18-T vector (Takara) and colonies were randomly selected and sent to the company for the sequencing. A pair of gene- specific primers designed from the putative 5′ and 3′ UTR sequences and were used to amplify the complete *NtMYB2* open reading frame (ORF). The sequence of forward and reverse primers are *NtMYB2* Forward: 5′-AATATGGGTAGGTCTCCTTGTTGTG-3′ and *NtMYB2* reverse: 5GATTACAGGACACGCAAA GTAAATCTA-3′ respectively. The PCR conditions were same as above. The amplified PCR product was purified using a gel extraction Kit (Omega, Norcross, GA, USA). The PCR product cloned into pMD18-T (Vector Takara, Dalian, China). The cloning product was transformed into *Escherichia coli* which were cultured on LB plate containing ampicillin at a final concentration of 50 mg/mL. Resistant colonies were confirmed by PCR using gene specific primers and M13 Primers and sent to the company (Biosune, Shangai, China) for the sequencing on both strands.

### 4.4. Expression Vector Construction

The ORF of *NtMYb2* was amplified and cloned into a plant expression vector pSAK277 containing the *CaMV 35S* promoter using In-Fusion^®^HD cloning kit (Clontech^®^ Laboratories, Inc., Palo Alto, CA, USA). The complete coding sequence of *NtMYB2* was amplified using a gene- specific forward primer with an *EcoRI* restriction site (TGGATCCAAAGAATTCAATATGGGTAGGTCTCCTTGT TGTG) and reverse gene-specific with primer with a *HindIII* restriction site (TACTCTCGAGAAGCTT GATTACAGGACACGCAAAGTAAATCTA). This overexpression constructed vector pSAK277-*NtMYB2* was introduced into the *Agrobacterium tumefaciens* GV3101 stain using the freeze-thaw method.

### 4.5. Sequence Analysis of NtMYB2

The theoretical *NtMYB2* coding sequence translation, isoelectric point (pI) and molecular weight of the *NtMYB2* was found by using expasy server (www.ExPASy.org). R2R3 MYB transcriptional factors (TFs) which are close related to *NtMYB2* used for building a phylogenetic tree. Phylogenetic trees were built using the neighbor joining tree method with partial deletion gap treatment. The bootstrap analysis with 1000 replicates was used to evaluate the tree nodes through MEGA5 software with default parameters. The sequences of amino acid of R2R3 MYB in other different plants were obtained from NCBI (Available online: www.ncbi.nlm.nih.gov/BLAST/) and TAIR database (Available online: https://www.arabidopsis.org). *NtMYB2* sequence was aligned with other deduced the amino acid sequence of other species using DNAMAN7.0 (Lynnon Corporation, San Ramon, CA, USA).

### 4.6. The Expression Pattern of NtMYB2 in Different Tissues of Chinese Narcissus

The *NtMYB2* gene expression in various tissues (Leaves, basal plate, flower petals and corona at different developmental stages) of Chinese narcissus was examined by real-time qRT-PCR. Total RNA was extracted using an Omega Total RNA Kit (Omega) from different tissues of Chinese Narcissus by following the instructions of plant extraction kit. The genomic DNA was removed from total RNA and cDNAs weresynthesized with the PrimeScript^TM^ RT reagent Kit with gDNA Eraser (Takara, Dalian, China). The synthesized cDNAs was diluted and used as template for qRT-PCR analysis. Quantitative Real time PCR was performed with a Light cyclar^®^480 real time PCR machine (Roche Diagnostics, Indianapolis, IN, USA) using the SYBR Green PCR master mix kit (Takara). The qRT-PCR condition was as follows: 95 °C for 30 s, 40 cycles of 95 °C for 3 s and 60 °C for 30 s and a default melt curve program. The relative expression level was estimated with the *NtActin* gene (Table S1) used as an internal standard. The comparative Ct method was carried out to estimate the gene expression level [58]. Three biological replicates and same technical replicate for each sample were carried out. Gene specific primers (Table 1) were used in Quantitative real time PCR analysis.

### 4.7. Transient Assays of NtMYB2 in Tobacco Leaves

The seeds of *Nicotiana tabacum* plants were grown in pots contain compost mix (1:1 compost: perlite and 3:1 compost: perlite for seed and plants respectively) and maintained in the glasshouse. The constructed pSAK277-*NtMYB2*, pSAK277-*StMYB*, pSAK277-*StbHLH* and pCAMBIA1301 vector was transformed into *Agrobacterium tumifaciens* stain GV3101 by electroporation. Potato *StMYB* (*StAN1*) and *StbHLH* were provided by Liu [45]. The *Agrobacterium* colonies were cultured in YEB media supplement with appropriate antibiotics and incubated at 28 °C at 190 rpm for overnight. The O.D._600_ was adjusted at 0.6–0.8 and checked by Spectrophotometer. *Agrobacterium* culture was centrifuged for 3 min at 3000 rpm in a microfuge tube and supernatant were removed. The obtained pellet was resuspended in agroinfiltration medium (MS salts 4.32 mg/L; 10 mM MES; 20 g/L sucrose; 200 mM acetosyringone) and left for 2–4 h at room temperature. Agroinfiltration suspension was injected into abaxial side of the young leaves of *Nicotiana tabacum. NtMYB2, StMYB*, *StMYB + NtMYB2* and *StMYB + StbHLH + NtMYB2* were used as treatments *StMYB* + *pCAMBIA1301* as control. Three biological replications are carried out in this transient assay. *Agrobacterium tumefaciens* infiltrated plants were keep at 25 °C in a growth chamber with a 10 h light and 14 h dark photoperiod for 4–6 days. After 5–6 days of agroinfiltration injection, change in color were observed in injected tobacco leaves. The photographs of infiltrated leaves were taken and the samples of injected leaves were collected and stored at −80 °C for further analysis and experiment.

### 4.8. Stable Transformation of Tobacco

Transformation of *Nicotiana tabacum* was carried out using the leaf disc method as previously described [59]. The positively transformed tobacco shoots were screened on MS medium using 100 mg/L kanamycin and 350 mg/L carbenicillin. Transgenic tobacco plants were confirmed by PCR assay from genomic DNA with the help of specific primers for *NtMYB4*. The generated kanamycin resistant tobacco plants were then transferred to a mixture of soil (1:1:1 of vermiculite, perlite and peat moss) for their adaptation under normal condition.

### 4.9. Extraction and Measurement of Total Anthocyanins and Phenolics 

Total anthocyanin content was extracted and measured in overexpression *NtMYB2* tobacco flowers according to the modified method reported previously [60]. The fresh flowers (0.5 g) were ground in liquid nitrogen and transferred into clean tube containing 6ml of methanol (1% HCl) and incubated overnight at 4 °C. Consequently, the tissues extracts were homogenized and centrifuged at 13,000 rpm (10 min, 4 °C). The absorbance of supernatants was determined at 530 nm and 657 nm. Total anthocyanin concentration was calculated as Q = (A530 − 0.25 × A657) × FW^−l^.

### 4.10. Statistical Analysis

Statistical analysis was carried out by one-way ANOVA. LSD values were calculated using *p* = 0.05 and used to compare treatment means.

## 5. Conclusions

In this study, a novel R2R3 MYB transcriptional factor, *NtMYB2*, with the potential to down-regulate the anthocyanin, flavonol and proanthocyanidin biosynthesis pathway, was identified. This MYB belongs to subgroup 4 R2R3 MYBs, which act as repressors of transcripts of key enzyme genes involved in flavonoid biosynthesis. Transcripts of *NtMYB2* are detected in flowers, leaves and basal plates in Chinese narcissus. Transcripts of *NtMYB2* are higher in flowers than other organs. Transient expression of *NtMYB2* in tobacco leaves and ectopic expression of *NtMYB2* in tobacco indicated that this MYB represses anthocyanin biosynthesis. The results from this study will be helpful to understand the regulating mechanism of anthocyanin and flavonoid biosynthesis in Narcissus and provide direction to improve the flower color and pattern in this species.

## Figures and Tables

**Table 1:** List of primers used for *Nt*MYB2 isolation and characterization.

Name	Forward Primer (5′ to 3′)	Reverse Primer (5′ to 3′)	Note
*GSP1*	ATGGGTAAACTATCTTCGGC	UPM (provided by company)	1st round of 3′-RACE
*GSP2*	GGAAGGACAGACAACGAGAT	UPM (provided by company)	2nd round of 3′-RACE
*NtMYB2*	AATATGGGTAGGTCTCCTTGTTGTG	GATTACAGGACACGCAAAGTAAATCTA	Cloning of full length of ORF
pSAK-NtMYB2	TGGATCCAAAGAATTCAATATGGGTAGGTCTCCTTGTTGTG	TACTCTCGAGAAGCTTGATTACAGGACACGCAAAGTAAATCTA	Vector construction
QRT	TGGGTAGGTCTCCTTGTTGTG	AAATTGCCTCTCTTGAGATCGG	qRT-PCR analysis

## Supplementary Materials

Supplementary materials can be found at.
