# Supplementary material for: Ectopic Overexpression of a Novel R2R3-MYB, NtMYB2 from Chinese Narcissus Represses Anthocyanin Biosynthesis in Tobacco
Source: Molecules. 2018 Mar 28;23(4):781. doi: 10.3390/molecules23040781 (PMC6017421; doi:10.3390/molecules23040781)
Supplement: Supplementary file 1 [file molecules-23-00781-s001.pdf]

Figure S 1. Confirmation of transgenic tobacco plants. Over expression *NtMYB2* lines (L-26, L33, and L41); WT, wild type. M represents for marker.

M L-26 L-33 L-41 WT

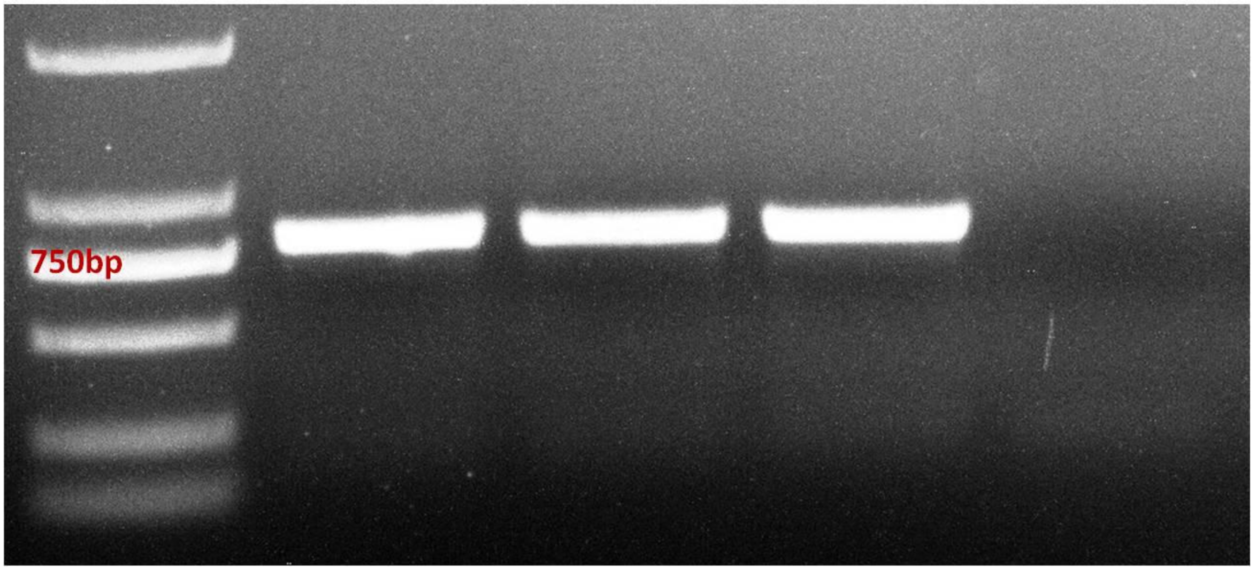

Table supplementary 1

Primers for real-time PCR analysis of tobacco genes of flavonoid biosynthesis pathway

Genes	GenBank ID	Forward primer (5' to 3')	Reverse primer (5' to 3')
NtCHS	AF311783	TTGTTCGAGCTTGTCTCTGC	AGCCCAGGAACATCTTTGAG
NtCHI	AB213651	GTCAGGCCATTGAAAAGCTC	CTAATCGTCAATGCCCCAAC
NtF3H	AB289450	CAAGGCATGTGTGGATATGG	TGTGTCGTTTCAGTCCAAGG
NtFLS	AB289451	GTCCACAACGTTGCATGGTG	CACAACTTCTCGCAGCCTC
NtDFR	EF421429	AACCAACAGTCAGGGGAATG	TTGGACATCGACAGTTCCAG
NtPAL	X78269	ATTGAGGTCATCCGTTCTGC	TCTGCAGCTCTTTCTGCATC
NtLAR	AM827419	TCAAGGTCCTTTACGCCATC	ACGAACCTGCTTCTCTTTGG
NtANR1	AM791704	CATTTGACTTTCCCAAACGC	ATTGGGCTTTTGAGTTGTGC
NtUFGT	FG627024	GAGTGCATTGGATGCCTTTT	CCAGCTCCATTAGGTCCTTG
NtActin	AY179605	AATGATCGGAATGGAAGCTG	TGGTACCACTGAGGACA
NtPAL	X78269	ATTGAGGTCATCCGTTCTGC	TCTGCAGCTCTTTCTGCATC
NtANS	AB289447	TGGCGTTGAAGCTCATACTG	GGAATTAGGCACACACTTTGC
Nt4CL	U50845	TCATTGACGAGGATGACGAG	TGGGATGGTTGAGAAGAAGG
